# Detecting overlapping protein complexes in PPI networks based on robustness

**DOI:** 10.1186/1477-5956-11-S1-S18

**Published:** 2013-11-07

**Authors:** Shuliang Wang, Fang Wu

**Affiliations:** 1 School of Software, Beijing Institute of Technology, 5 South Zhongguancun Street, Haidian District, Beijing 100081, China; 2 The Integrated Information System Research Center, Institute of Automation, Chinese Academy of Sciences, 95 Zhongguancun East Road, Haidian District, Beijing 100190, China

## Abstract

**Background:**

Recently, large data sets of protein-protein interactions (PPI) which can be modeled as PPI networks are generated through high-throughput methods. And locally dense regions in PPI networks are very likely to be protein complexes. Since protein complexes play a key role in many biological processes, detecting protein complexes in PPI networks is one of important tasks in post-genomic era. However, PPI networks are often incomplete and noisy, which builds barriers to mining protein complexes.

**Results:**

We propose a new and effective algorithm based on robustness to detect overlapping clusters as protein complexes in PPI networks. And in order to improve the accuracy of resulting clusters, our algorithm tries to reduce bad effects brought by noise in PPI networks. And in our algorithm, each new cluster begins from a seed and is expanded through adding qualified nodes from the cluster's neighbourhood nodes. Besides, in our algorithm, a new distance measurement method between a cluster *K *and a node in the neighbours of *K *is proposed as well. The performance of our algorithm is evaluated by applying it on two PPI networks which are Gavin network and Database of Interacting Proteins (DIP). The results show that our algorithm is better than Markov clustering algorithm (MCL), Clique Percolation method (CPM) and core-attachment based method (CoAch) in terms of F-measure, co-localization and Gene Ontology (GO) semantic similarity.

**Conclusions:**

Our algorithm detects locally dense regions or clusters as protein complexes. The results show that protein complexes generated by our algorithm have better quality than those generated by some previous classic methods. Therefore, our algorithm is effective and useful.

## Background

Nowadays, with the rapid development of advanced proteomics technologies such as mass spectrometry [1], microarrays [2] and phage display [3], large data sets of PPIs are generated. And a common way to analyze these data sets is to portray them as PPI networks. In the post-genomic era, understanding PPI networks is an important task. It is suggested that locally dense regions in PPI networks are likely to be protein complexes. Protein complexes involve genes or proteins which participate in common fundamental biological processes [4]. Since protein complexes play a vital role in the cellular organization and function, detecting protein complexes in PPI networks becomes a burning issue which attracts many researchers.

In order to solve the problem of detecting protein complexes in PPI networks, many researchers have proposed a lot of computational approaches. The method proposed by Spirin and Mirny [5] in 2003 tried to find all fully connected subgraphs (cliques) as protein complexes by complete enumeration. However, identifying all cliques in PPI networks is an NP-complete problem and clique is a very restrictive condition which leaves out many potential protein complexes. In order to improve this method, CPM [6] mined k-clique percolation clusters as protein complexes. Although CPM can detect more protein complexes than [5], it still omits a lot of protein complexes because many protein complexes are not k-clique percolation clusters. And Molecular Complex Detection (MCODE) [7] proposed by Bader and Hogue was a kind of density-based methods. Based on node weight, MCODE detects locally dense regions as protein complexes. In MCODE algorithm, node weight is assigned to each node in the network according to its local neighbourhood density. In general, MCODE is an effective approach, but it detects only a few protein complexes in PPI networks. Besides, Highly Connected Subgraph method (HCS) [8], one of graph partition-based methods, used minimum cuts to separate a PPI network into several subgraphs. The resulting subgraphs are regarded as protein complexes as long as they satisfy a specified density threshold. And MCL [9], another kind of graph partition-based methods, produced highly connected subnetworks as protein complexes through random walks. Though HCS and MCL are fast, they do not support mining overlapping protein complexes. However, in reality, many protein complexes do share proteins. In addition, Restricted Neighborhood Search Clustering Algorithm (RNSC) [10] which was a cost-based local search algorithm partitioned PPI networks through minimizing a cost function. Since RNSC is a randomized algorithm, the same input data will make RNSC produce different results. And in order to improve the accuracy of clustering results, RNSC uses a filtering step. This step needs information of functional homogeneity, which is a shortcoming of RNSC. And recently, CoAch [11] has been proposed according to the idea in [12] that a protein complex generally includes a core and several attachments. It first detects cores from each nodes' neighbourhood graph and then expands each core by adding nodes which connect more than half of nodes in the core.

Kelly et al. [13] found that many alternative paths often exist between functionally associated proteins since they contribute a lot to the robustness of biological signals' transmission. Therefore, protein complexes should have the characteristic of robustness. As an expansion upon the proceedings version [14], we propose a novel algorithm to mine overlapping protein complexes on the basis of robustness. In our algorithm, a seed is firstly selected as a new cluster and expands the cluster by adding qualified nodes from the cluster's neighbourhood nodes. Since many existing PPI networks are incomplete and noisy, each edge's weight in resulting clusters should satisfy a specified threshold. The weight of an edge in PPI network is assigned on the basis of the similarity of nodes at the end of the edge. Besides, resulting clusters should have relatively high robustness for trying to find out reliable protein complexes. Moreover, we present a new distance measurement method between a cluster *K *and a node in the neighbours of *K *as well. In order to estimate the performance, our algorithm is compared with MCL, CPM and CoAch. The results show our algorithm has an improvement in terms of F-measure, co-localization and GO semantic similarity.

## Methods

### Terminology

A PPI network can be modeled as an undirected simple graph *G*=(*V, E*), in which *V *represents the set of nodes (proteins) and *E *represents the set of edges (protein interactions) in the network. Here, self-interactions (loops) and multiple edges between the same pair of nodes are not considered. Before detail description of our algorithm, some terminologies used in the following algorithm section are presented as follows.

#### Definition 1

In our algorithm, the weight *w_uv _*of edge (*u, v*) ∈ *E *is defined as the similarity between *u *and *v*. It is obvious that two nodes with an edge between them belong to the same cluster if they have high similarity. The similarity between *u *and *v *is measured by Jaccard's coefficient [2]. Jaccard's coefficient adopts the proportion of common neighbours of two nodes in all distinct neighbours of these nodes to measure node similarity in complex networks. Obviously, the more common neighbours two nodes share, the higher similarity these nodes have. Therefore, the edge weight *w_uv _*is represented by Equation (1).

(1) $$w_{uv}=\frac{\text{Γ}\left(u\right)\cap \text{Γ}\left(v\right)}{\text{Γ}\left(u\right)\cup \text{Γ}\left(v\right)}$$

where, Γ(*u*) and Γ(*v*) are neighbours of *u *and *v *respectively. Γ(*u*) ∩ Γ(*v*) represents all common neighbours of *u *and *v*, and Γ(*u*) ∪ Γ(*v*) represents all distinct neighbours of *u *and *v*. In our algorithm, edge weight is used to guarantee that in the same cluster every pair of nodes with an edge between them should have relatively high similarity.

#### Definition 2

The neighbourhood graph [11] of v∈V consists of *v*, all its neighbours and the edges among them. It is defined as $G_{v}=\left(V^{\prime },E^{\prime }\right),$in which $V^{\prime }=\left\{v\right\}\cup \left\{u|u\in V,\left(u,v\right)\in E\right\},$ and $E^{\prime }=\left\{\left(u_{i},u_{j}\right)|\left(u_{i},u_{j}\right)\in E,u_{i},u_{j}\in V^{\prime }\right\}.$

#### Definition 3

In *G_v_*, there are some nodes with degree 1 that only have connections with *v *and the connections among these nodes are often false positive according to topological reliability measures described in [15-17]. So all nodes with degree 1 and corresponding edges are removed from *G_v _*. The remaining subgraph of *G_v _*is marked as ${G_{v}}^{\prime }$. In our algorithm, the node weight *w_v _*of node v∈V in PPI networks is the average degree of all nodes in ${G_{v}}^{\prime }$. It is represented by Equation (2).

(2) $$w_{v}=\frac{\underset{u\in V^{\prime \prime }}{\sum }\text{deg}\left(u\right)}{\left|V^{\prime \prime }\right|}$$

where, *V" *is the set of nodes in ${G_{v}}^{\prime }\;.|V^{\prime \prime }|$ is the number of nodes in ${G_{v}}^{\prime }$. And deg(*u*) is the degree of a node *u *∈ *V" *in ${G_{v}}^{\prime }$. In our algorithm, the weight *w_v _*of a node *v *∈ *V *is used in the step of seed chosen. If *w_v _*is big enough, *v *has many neighbours in ${G_{v}}^{\prime }$ and ${G_{v}}^{\prime }$ is a densely connected region.

#### Definition 4

*N_k _*of a cluster *K *= (*V_K_, E_K_*) is a set of all nodes which do not belong to *K *but have edges connected to nodes in *K*. Czekanovski-Dice distance [18] has been used to measure the functional distance of two nodes in complex networks. In this paper, we modify the Czekanovski-Dice distance for measuring the functional distance *d*(*v*, K) between a cluster *K *( *K*= (*V_k_, E_k_*)) and a node *v *(*v *∈ *N_k_*).

(3) $$d\left(v,K\right)=\frac{|K|-m}{\left|K\right|}+\frac{OUT\left(K\right)+OUT\left(v\right)}{\text{deg}\left(K\right)+\text{deg}\left(v\right)-2m+|K|}$$

where, | *K *| is the number of nodes in *K *. *m *is the number of all edges between *v *and any node in *K *. deg(*K*) is the total external degree of *K *. *OUT*(*K*) is the number of edges connecting any node in *K *with nodes outside *K *which do not include *v *and common neighbours of *K *and *v *. And *OUT*(*v*) is the number of all edges connecting *v *with nodes which do not include all nodes in *K *and common neighbours of *K *and *v *. Equation (3) defines the function distance of *K *and *v *according to both direct connections and common neighbours of *K *and $v\;.\frac{|K|-m}{\left|K\right|}$ considers the influence of direct connections between *K *and *v *on the function distance. $\frac{OUT\left(K\right)+OUT\left(v\right)}{\text{deg}\left(K\right)+\text{deg}\left(v\right)-2m+|K|}$ considers the influence of common neighbours of *K *and *v *on the function distance. And | *K *| is an adjustment factor.

#### Definition 5

Given a cluster *K *= (*V_K_, E_K_*), we remove the node with the highest degree and relative edges in *K*. Then, in the remaining graph of *K*, we continue to remove the node with the highest degree and relative edges. The above step is iterated until the remaining graph of *K *is not connected or contains no nodes. Obviously, the number of nodes removed can show the robustness degree of *K*. Therefore, the robustness degree of *K *can be defined in Equation (4).

(4) $$R_{K}=\frac{n}{\left|K\right|}$$

Where, n is the number of nodes removed until the remaining graph of *K* is not connected or contains no nodes. And |K| is the number of all nodes in *K* before removing operation begins.

#### Definition 6

The neighbour affinity *NA*(*A, B*) [18] of two clusters i.e. *A *= (*V_A_, E_A_*) and *B *= (*V_B_, E_B_*) is defined in Equation (5), for measuring their overlapping degree.

(5) $$NA\left(A,B\right)=\frac{|V_{A}\cap V_{B}{|}^{2}}{\left|V_{A}|\times |V_{B}\right|}$$

### Algorithm

**Figure 1:**
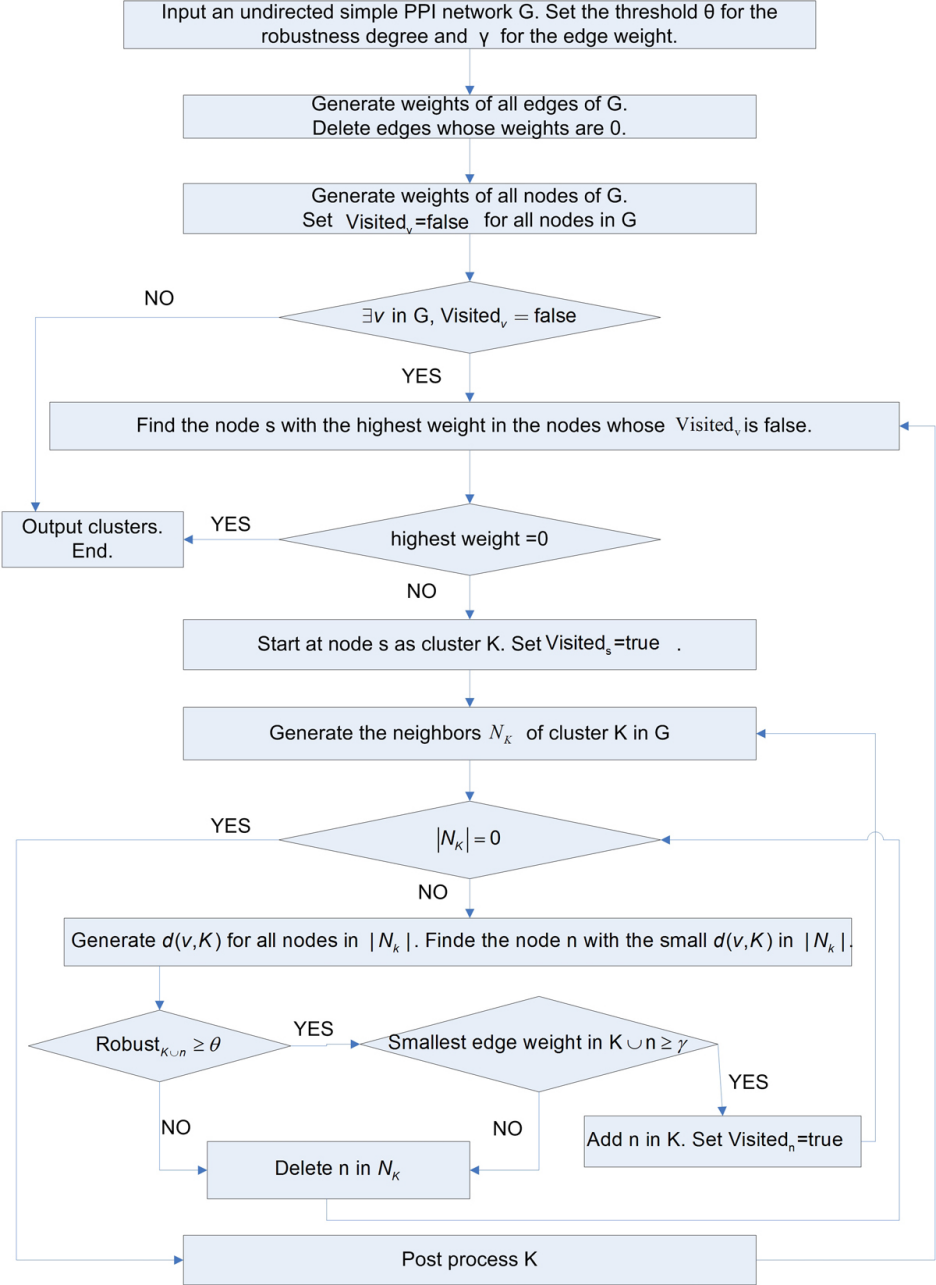
**The flowchart of our algorithm**. Figure 1 demonstrates concrete steps of our algorithm.

In this section, main steps of our algorithm are discussed in turns. To explain it in more detail, Figure 1 shows the flowchart.

Our algorithm includes five main steps which are Input & initialization, Termination, Seed chosen, Cluster formation and Post processing. Each step will be discussed below.

#### Input & initialization

The input to our algorithm includes an undirected simple PPI network and two parameters which are the value *θ *of minimum robustness degree allowed for generated clusters, and the value *γ *of minimum edge weight allowed in clusters.

In the step of initialization, all edges in *G *whose edge weights are 0 are deleted since they often have low reliability. In addition, set *Visited_v _*= false for every node in the PPI network. The *Visited_v _*attribute of a node *v *records whether *v *has been assigned to any generated cluster or not.

#### Termination

Our algorithm is terminated when following circumstances appear: (1) all nodes in the network are assigned to generated clusters, and (2) the highest node weight in all node weights of nodes which are not assigned to any cluster is 0, which means all nodes which do not belong to any cluster are isolated. Then, generated clusters which have no less than 3 nodes are printed out.

#### Seed chosen

Each cluster starts at a node which is called seed. In the area of detecting protein complexes in PPI networks, seed should have following characteristics. Direct neighbours of seed should be as many as possible and neighbourhood graph of seed should be as densely connected as possible.

In our algorithm, the measurement method of node weight in (2) satisfies requirements of seed selection. Therefore, (2) is used in this step. In G, the node with the highest node weight among all nodes which are not assigned to any generated cluster is selected as seed. And the *Visited_v _*attribute of seed is set to true.

#### Cluster formation

A cluster *K * starts at seed and then grows gradually by adding nodes one by one from neighbours of *K *which are included in the set *N_K _*. At each time, the node with the highest priority in *N_K _*is taken into consideration. The priority depends on the functional distance *d *(*v*, K) between a node *v *and a cluster *K *in Equation (3). The smaller *d*(*v*, K) is, the higher priority *v *has.

Before adding the node with the highest priority to cluster *K*, two conditions must be checked. First, we make sure that the node's addition in *K *does not cause the robustness degree of *K *to fall below *θ *which is the minimum robustness degree allowed for generated clusters. Second, we guarantee that the node's addition in *K *does not lead the minimum edge weight in *K *to fall below *γ *which is the allowed minimum edge weight in generated clusters. These two conditions guarantee that resulting clusters are densely connected. If any of the above conditions is not satisfied, we delete the node in *N_K_*. Then, we continue to check whether the node with the highest priority in the updated *N_K _*satisfies the two conditions or not.

#### Post processing

Assume that *CK *is the set of all currently produced clusters and *K *is a newly detected cluster. Before *K *is added in *CK *, neighbour affinities between *K *and any cluster in *CK *are calculated and the cluster *K_M _*which has the largest neighbour affinity with *K *in *CK *is discovered. If the neighbour affinity *NA *(*K, K_M _*) is greater than or equal to 0.5, *K_M _*and *K *will be merged. Otherwise, *K *will be added in *CK *as an independent cluster. This step not only allows generated clusters share common nodes but also prevents generated clusters from having too high overlapping degrees with each other.

## Results

### Datasets

In this paper, Gavin [12] dataset and DIP [19] which are produced through high-throughput technology are used to validate our algorithm. In Gavin dataset, there are 1430 proteins and 6532 interactions. And in DIP dataset, there are 4,928 proteins and 17,201 interactions.

In order to evaluate the quality of predicted protein complexes, a benchmark set presented in [20] is obtained form MIPS [21], Aloy et al. [22] and Saccharomyces Genome Database (SGD) [23] according to GO notations. In this benchmark set, 428 protein complexes are included.

### Evaluation criteria

#### Precision, Recall and F-measure

Assume *p *is a predicted protein complex and *b *is a real protein complex in the benchmark. Whether *p *and *b *match to each other or not is determined by the neighbour affinity *NA*( *p, b*). If *NA *(*p, b*). ≥ *w, p *and *b *are thought to be matched. Usually, it's a common way to set *w* to 0.2.

Assume *B *is the benchmark set and *P *is the set of predicted protein complexes. The number of predicted protein complexes which match at least one real protein complex in *B *is marked as *N_cp_*. And the number of real protein complexes which match at least one predicted protein complex in *P *is marked as *N_cb_*. Then, two kinds of evaluation criteria which are precision and recall are defined in Equation (6) and Equation (7) respectively. F-measure defined in Equation (8) is the harmonic mean of precision and recall. It is an important kind of criteria to evaluate the quality of predicted protein complexes.

(6) $$\text{Precision}=\frac{N_{cp}}{\left|P\right|}$$

(7) $$\text{Recall}=\frac{N_{cb}}{\left|B\right|}$$

(8) $$\text{F - measure = }\frac{\text{2x Precision Recall}}{\text{Precision + Recall}}$$

#### Co-localization

Although precise, recall and F-measure are very popular evaluation criteria, they are not alone. Biological relevance is also widely used in evaluating the quality of predicted protein complexes. One way to evaluate the biological relevance is co-localization score. Since proteins in the same protein complex have a tendency to share common functions, they tend to be at the same localization. And in a protein complex, different localizations may exist. Thus, the Co-localization score of a protein complex *C *is measured by the maximal proportion of proteins locating at the same localization in *C *[20]. Assume the sum of proteins in *C *which share location *i is *| *V_i _*| and the total number of proteins in *C is *| *V *|. Thus, the Co-localization score of *C *can be defined in Equation (9).

(9) $$Co-localization\left(C\right)=\text{max}\left(\frac{\left|V_{i}\right|}{\left|V\right|}\right)$$

Obviously, the higher co-localization score is, the higher functional similarity between proteins in the same complex is. Therefore, co-localization score is a good way to evaluate the quality of predicted protein complexes.

#### GO semantic similarity

Comparing GO terms associated with proteins in a protein complex is another indicator of biological relevance. According to GO information, a novel method to measure GO semantic similarity is proposed in [24]. In [24], for a protein complex, GO semantic similarity score is defined as the average of semantic similarity scores of all protein pairs within the protein complex. And for a set of protein complexes, GO semantic similarity score is defined as the geometric mean of scores of three ontologies which are "cellular component", "biological process" and "molecular function" ontology respectively. These three ontologies scores are obtained from GO semantic similarity scores of all complexes in the set. Therefore, the higher GO semantic similarity score of a set of protein complexes is, the better the quality of the set of predicted protein complexes is.

### Complex set comparative evaluation

In this section, the above three categories of evaluation criteria are used to compare the performance of our algorithm with three classic methods which are MCL, CPM and CoAch.

#### Precision, Recall and F-measure Comparison

Table 1 and Table 2 show comparisons of MCL, CPM, CoAch and our algorithm using Gavin and DIP dataset. In these tables, *N_cp _*is the number of predicted protein complexes matching at least one real protein complex in *B *and | *P *| is the total number of predicted complexes.

**Table 1:** The comparison of various algorithms using Gavin dataset.

Algorithm	*N_cp_*	| *P *|	Precision(%)	Recall(%)	F-measure(%)
MCL	103	232	44.40	42.76	43.56
CPM	54	98	55.10	20.79	30.19
CoAch	178	326	54.60	41.36	47.06
Our algorithm	116	185	62.70	38.32	47.56

Table 1 shows the comparison of MCL, CPM, CoAch and our algorithm using Gavin dataset. The comparison is based on the number of predicted complexes, the number of predicted complexes which match at least one known protein complex, precision, recall and F-measure.

**Table 2:** The comparison of various algorithms using DIP dataset

Algorithm	*N_cp_*	| *P *|	Precision(%)	Recall(%)	F-measure(%)
MCL	212	1246	17.01	59.81	26.49
CPM	84	245	34.29	25.93	29.53
CoAch	286	747	38.29	58.18	46.18
Our algorithm	192	422	45.50	54.21	49.17

Table 2 demonstrates the comparison of MCL, CPM, CoAch and our algorithm using DIP dataset. The comparison is based on the number of predicted complexes, the number of predicted complexes which match at least one known protein complex, precision, recall and F-measure.

From Table 1, it is obvious that our algorithm has the highest precision and the third highest recall among the four algorithms. And in Table 1, F-measure of our algorithm is the highest. Similarly, in Table 2, it can be found that our algorithm achieves the highest precision and the third highest recall among the four algorithms. Also, F-measure of our algorithm in Table 2 is the highest.

Recall of our algorithm is not very high because the number of protein complexes predicted by our algorithm is not large. And since MCL and CoAch predict large number of protein complexes, they achieve higher recall than our algorithm. The reason that our algorithm predicts a limited number of protein complexes is that our algorithm tries to use robustness degree and edge weight to find reliable protein complexes in incomplete and noisy PPI networks. Thus, protein complexes predicted by our algorithm have very high precision at the cost of not high recall. Although recall is not high, our algorithm still obtains the highest F-measure on both Gavin and DIP dataset. And as F-measure takes both precision and recall into consideration, it can be found that protein complexes detected by our algorithm have good quality.

#### Co-localization similarity

The localization dataset published by Huh et al. [25] is used in the calculation of co-localization score. It is often used in evaluating the quality of protein complexes. And in this dataset, 75% of yeast proteome are classified into 22 distinct sub-cellular locations. In addition, ProCope [24], a popular tool which offers easy access to different ways to predict and evaluate protein complexes, is used to calculate the co-localization score.

**Figure 2:**
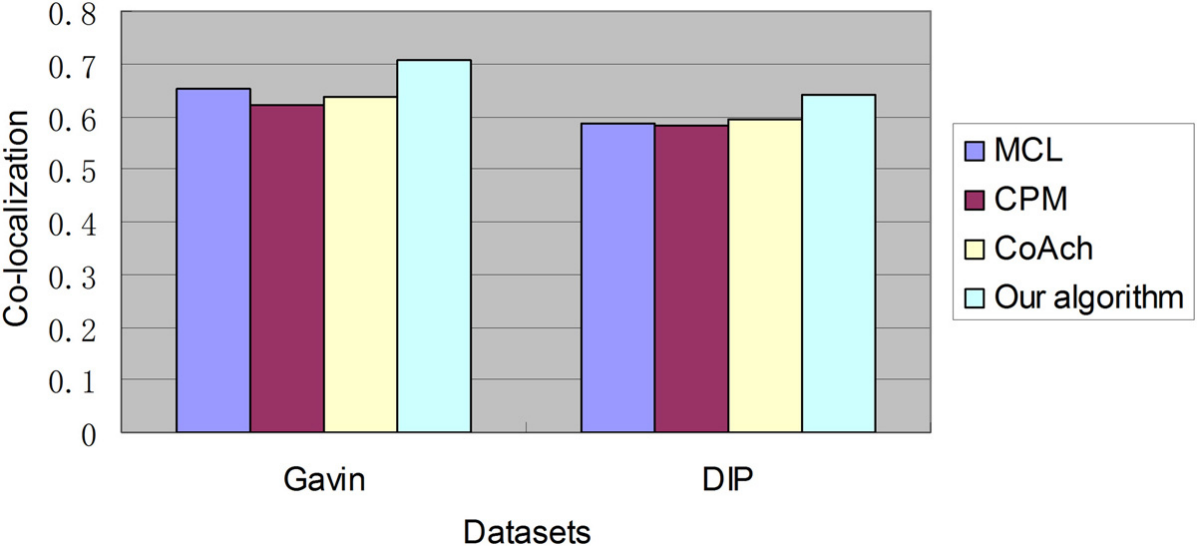
**The co-localization similarity comparisons on Gavin and DIP dataset**. Figure 2 shows co-localization similarity results of MCL, CPM, CoAch and our algorithm on Gavin and DIP dataset.

Figure 2 shows co-localization scores of protein complexes detected through four algorithms on Gavin and DIP dataset. The co-localization score of our algorithm on Gavin dataset is 0.707, which is higher than 0.653 obtained by MCL, 0.621 obtained by CPM and 0.636 obtained by CoAch. And the co-localization score of our algorithm on DIP dataset is 0.640, which is higher than 0.587 obtained by MCL, 0.583 obtained by CPM and 0.595 obtained by CoAch.

Since proteins within a complex tend to be at the same sub-cellular locations, co-localization score can be used to evaluate the biological relevance of predicted protein complexes. And high co-localization score means high biological relevance of predicted protein complexes. Thus, as our algorithm achieve highest scores on both Gavin and DIP dataset, it is obvious that protein complexes detected by our algorithm have relatively high quality from the biological view.

#### GO semantic similarity

The similarity of two different GO terms is determined by their most recent common ancestors in the ontology structure. And the similarity of GO terms related to proteins in a protein complex can demonstrate the biological relevance within the complex. In this section, we also use GO semantic similarity score which can show the similarity of proteins within a complex to evaluate the quality of predicted protein complexes. We still make use of ProCope to calculate the GO semantic similarity score.

**Figure 3:**
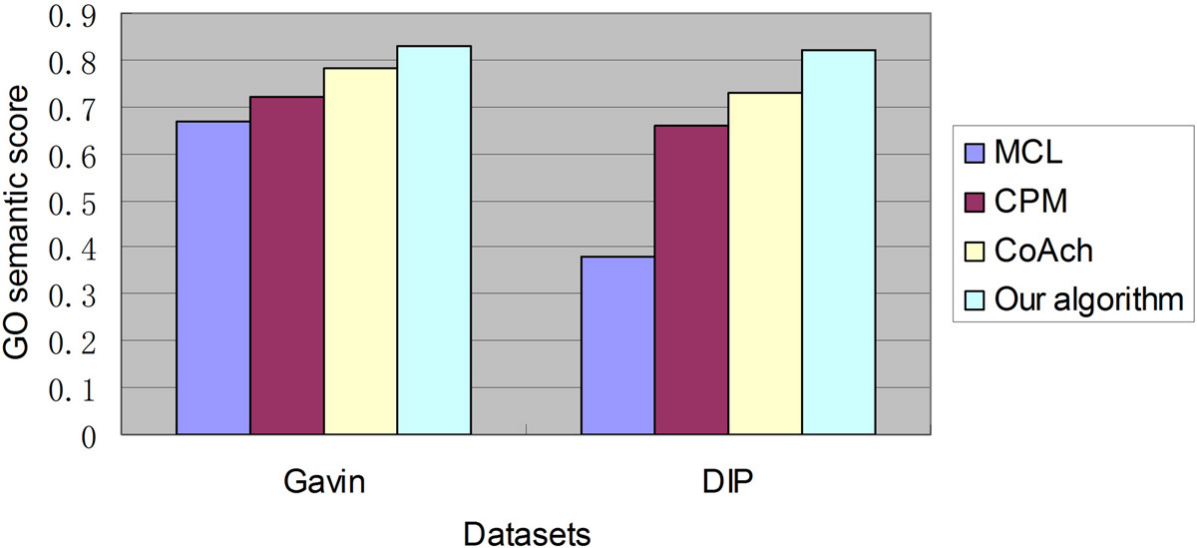
**The GO semantic similarity comparisons on Gavin and DIP dataset**. Figure 3 shows GO semantic similarity results of MCL, CPM, CoAch and our algorithm on Gavin and DIP dataset.

Figure 3 shows results of comparisons of GO semantic similarity scores achieved through using the four algorithms on Gavin and DIP dataset. The GO semantic similarity score of our algorithm on Gavin dataset is 0.83, which is higher than 0.67 obtained by MCL, 0.72 obtained by CPM and 0.78 obtained by CoAch. And The GO semantic similarity score of our algorithm on DIP dataset is 0.82, which is higher than 0.38 obtained by MCL, 0.66 obtained by CPM and 0.73 obtained by CoAch.

As high GO semantic score means high biological relevance of predicted complexes, our algorithm which achieves the highest GO semantic scores on both Gavin and DIP dataset can detect protein complexes with relatively good quality.

## Conclusions

In this paper, a novel algorithm based on robustness to detect overlapping protein complexes is proposed. First, we present a method to measure the functional distance between a cluster and a node in the neighbours of the cluster. Then, we explain our algorithm in details. Our algorithm is a dense-based approach and tries to find reliable protein complexes in PPI networks. Finally, three categories of evaluation criteria are used to compare the performance of our algorithm with MCL, CPM and CoAch. The results show that our algorithm is better than MCL, CPM and CoAch in terms of F-measure, co-localization and GO semantic similarity, which means our algorithm can discover protein complexes with good quality. Therefore, our algorithm is effective and can be helpful in the future biological study.

## Competing interests

The authors declare that they have no competing interests.

## Authors' contributions

The algorithm is designed by SW and FW. And FW fulfills the algorithm and conducts related experiments. Both SW and FW have read and revised this paper.

## Declarations

The publication costs for this article were funded by the corresponding author.

This article has been published as part of *Proteome Science *Volume 11 Supplement 1, 2013: Selected articles from the IEEE International Conference on Bioinformatics and Biomedicine 2012: Proteome Science. The full contents of the supplement are available online at http://www.proteomesci.com/supplements/11/S1.
